# Circadian Regulation of Hippocampal-Dependent Memory: Circuits, Synapses, and Molecular Mechanisms

**DOI:** 10.1155/2018/7292540

**Published:** 2018-02-08

**Authors:** Kaitlin H. Snider, Kyle A. Sullivan, Karl Obrietan

**Affiliations:** Department of Neuroscience, Ohio State University, Columbus, OH 43210, USA

## Abstract

Circadian modulation of learning and memory efficiency is an evolutionarily conserved phenomenon, occurring in organisms ranging from invertebrates to higher mammalian species, including humans. While the suprachiasmatic nucleus (SCN) of the hypothalamus functions as the master mammalian pacemaker, recent evidence suggests that forebrain regions, including the hippocampus, exhibit oscillatory capacity. This finding, as well as work on the cellular signaling events that underlie learning and memory, has opened promising new avenues of investigation into the precise cellular, molecular, and circuit-based mechanisms by which clock timing impacts plasticity and cognition. In this review, we examine the complex molecular relationship between clock timing and memory, with a focus on hippocampal-dependent tasks. We evaluate how the dysregulation of circadian timing, both at the level of the SCN and at the level of ancillary forebrain clocks, affects learning and memory. Further, we discuss experimentally validated intracellular signaling pathways (e.g., ERK/MAPK and GSK3*β*) and potential cellular signaling mechanisms by which the clock affects learning and memory formation. Finally, we examine how long-term potentiation (LTP), a synaptic process critical to the establishment of several forms of memory, is regulated by clock-gated processes.

## 1. Introduction

Forty-five years ago, Davies et al. [1] first demonstrated that the efficiency of both learning and memory is modulated as a function of the time-of-day. Using a passive avoidance task (where animals learn to avoid a mild foot shock by remaining in the lighted side of a test chamber), they demonstrated that rats learned and remembered the task better during the day than during the night. In the decades since, numerous labs have confirmed that learning and memory are gated over the diurnal cycle [1–19]. Further, many of these diurnally regulated effects on learning and memory efficiency persist even when external time cues are eliminated [4, 12, 17, 18, 20]; thus this gating process is governed by inherent circadian timekeeping capacity. Circadian variation in memory is seen across phyla, from Aplysia [21] and fruit flies [22] to mice [4] and humans [23]. Furthermore, alterations in clock timing (whether resulting from jet lag [24, 25] or shift work [26, 27]) cause cognitive deficits. Likewise, cognitive impairment and dysregulation of the circadian timing system are comorbid (and possibly interrelated) features of many neurodegenerative disorders [28, 29], including Alzheimer's [30, 31], Parkinson's [32–34], and Huntington's disease [35–37]. These findings suggest that inherent circadian timekeeping capacity has a profound influence on the cellular and systems-based circuitry that underlies memory formation. As such, a better understanding of the mechanisms by which the circadian clock modulates cognition has wide-ranging implications for human health, disease progression, and overall quality of life.

In this review, we focus on circadian modulation of hippocampal-dependent forms of learning and memory. To this end, our discussion will be focused on hippocampus-dependent forms of spatial and contextual memory [38]. Importantly, both the circuits and many of the molecular mechanisms that underlie spatial and contextual memory are well characterized [39–41], and as such, the effects of the clock timing system can be examined within a functionally relevant cellular context [20, 42–44]. Further, we will present an extensive overview of the existing hypotheses on the mechanisms by which circadian rhythms modulate memory efficiency, as well as comment on broader implications of these hypotheses. We will begin by briefly discussing several hippocampal forms of learning and memory, describing the assays used to examine these processes, and reviewing work that places these memory processes into a circadian context.

In rodent model systems, circadian modulation of a wide array of hippocampal-dependent memory processes has been reported (for a summary see Table 1). Notably, while the learning and memory assays discussed in this review require the hippocampus, the level of this hippocampal dependence varies from assay to assay. Some assays (e.g., novel object location) are almost exclusively dependent on the hippocampus [45], while others (contextual fear conditioning or novel object recognition) also involve additional brain regions such as the amygdala and perirhinal cortex, respectively [45–47]. Generally, memory processes can be divided by mechanism and time scale into working or short-term memory, intermediate-term memory, and long-term memory (which requires both acquisition and retrieval). Working memory is a short-term information storage system that functions from seconds to a few minutes. It is dependent primarily on persistent neuronal activity (i.e., continued activation of a neuron or network after cessation of the stimulus [48]) and, at a cellular level, the trafficking of glutamate receptors to the synaptic membrane [49–52]. The radial arm maze is one example of a behavioral task used to measure hippocampal-dependent working memory. In this task, mice explore a maze with eight “arms” and a single food reward on each arm. Since an arm that has been visited no longer has a food reward, reentering a previously entered arm constitutes an error. This task provides a robust assessment of visuospatial learning in rodents, as arm discrimination is primarily dictated by visual cues [53], whereas olfactory cues are crucial only when the test is conducted in total darkness [54]. Using this assay, several studies have shown that working memory efficiency is modulated by the time-of-day. Along these lines, Rawashdeh et al. [55] reported significantly better performance (i.e., fewer errors) on the radial arm maze during the day compared to the night. Interestingly, Hauber and Bareiss [3] found better working memory performance on the radial arm maze during the night. There are several potential explanations for these disparate findings, including species differences (Hauber and Bareiss used rats, while Rawashdeh et al. used mice), experimental protocol differences (Hauber and Bareiss found improvement at night following 5 days of habituation and 10 days of training, while Rawashdeh et al. found improvement during the day following 2 days of habituation and 5 days of training), lighting conditions (both studies tested rodents under dim red light (<10 lux) during night but used different intensities of white light during day), or the time at which training occurred (Rawashdeh et al. tested rodents at ZT2 (day) and ZT14 (night), while Hauber and Bareiss tested animals during a period of ZT5-10 for day and ZT19-24 for night).

In contrast with working memory, intermediate-term memory is a process with a time scale of several minutes to a few hours. Mechanistically, intermediate-term memory shifts away from the persistent neuronal activity process that underlies working memory and towards a stronger dependence on mechanisms downstream of neuronal activation, including persistent activation of protein kinases and inducible mRNA translation (but not gene transcription) [39, 56, 57]. Novel object location is a test of spatial intermediate-term memory that is gated as a function of the time-of-day. For the novel object location task, the animal first explores two objects placed in reference to a visuospatial arena (shapes on the walls of the arena differentiate each direction). Thirty to sixty minutes later, the animal is returned to the arena; meanwhile, one of the objects has been moved. More time spent exploring the object which has been moved (i.e., the object in a novel location) indicates recollection of the initial spatial location. Both Takahashi et al. [14] and Snider et al. [20] found that animals were only able to discriminate between the novel object location and the familiar object location during the night (no discrimination was observed during the day); as overall exploration was the same at all times tested, this effect did not depend on time-of-day differences in the overall amount of exploration of the objects.

In contrast to intermediate-term memory, long-term memory involves two distinct processes. The initial process of acquisition (the coding of an experience) depends on gene transcription, de novo protein translation, and alterations in neuronal connectivity but not on sustained neuronal activity [58–61]. By contrast, the second process, retrieval (accessing the coded experience), is regulated by synaptic strength and glutamate receptor trafficking at the time that the memory is accessed [58, 62, 63] and does not require protein translation or gene transcription [39, 58]. One of the best characterized forms of hippocampal-dependent long-term memory is contextual fear conditioning. Contextual fear conditioning involves first administering a mild electric foot shock to an animal in a visuospatial context. When the animal is returned to that context (days or even weeks later), freezing behavior indicates an association of the shock with the visuospatial context. Several studies have shown that contextual fear conditioning is gated as a function of the time-of-day. Along these lines, Chaudhury and Colwell [4] and Eckel-Mahan et al. [18] showed that long-term contextual fear conditioning memory is more efficient during the day than the night. Interestingly, Valentinuzzi et al. [19] observed more efficient contextual fear conditioning memory during the night. Possible explanations for these diverging time-of-day dependent results include differing lighting conditions (Chaudhury and Colwell and Eckel-Mahan et al. entrained to a 12 h/12 h light/dark cycle, while Valentinuzzi et al. used constant dim green light with a skeleton white light photoperiod), differing measurement methods (Chaudhury and Colwell and Eckel-Mahan et al. used seconds freezing, while Valentinuzzi et al. used latency to a beam break), and relation to baseline behavior (Chaudhury and Colwell and Eckel-Mahan et al. examined overall percent freezing, while Valentinuzzi et al. normalized each animal's beam breaks by subtracting baseline beam breaks).

Together, these studies reveal important and fundamental features of the functional effects of circadian modulation on memory processes. However, a key issue that has not yet been fully examined in rodent models relates to the ability of assays to distinguish time-of-day effects mediated by encoding from time-of-day effects that are dependent on retrieval. Along these lines, in most rodent studies of time-of-day modulation of long-term memory, the learning experience and the retrieval test are conducted at the same time-of-day (e.g., learn at early day and test at early day). Thus, it is not possible to determine whether differences are due to enhanced acquisition or enhanced retrieval. For example, a task that is better during the day could be due to better acquisition of the task; or it could be that the animals learned the task equally well at both times but retrieved the memory more efficiently during the day. Since the molecular mechanisms of encoding and retrieval are distinct, clarifying which memory process is primarily impacted by circadian rhythms should be a top priority. Work in invertebrate models [64, 65], as well as a handful of current studies in rodents [4, 42], has made strides in this direction: they designed tests of long-term memory efficiency such that the memory is retrieved at a time-of-day that is distinct from the time-of-day of the initial learning experience. Future studies examining the circadian regulation of long-term memory should seek to build on their work.

Finally, it is also important to note that time-of-day differences in performance on hippocampal-dependent tasks may be confounded by interactions with sleep/wake cycles and the circadian clock. Sleep itself is a powerful modulator of memory [66, 67], and studies of nocturnal rodents involving a day (sleep phase) time point will certainly risk sleep disruption. Moreover, both light during the night (active) phase [68] and forced or novelty-induced activity during the day (sleep) phase [69] are capable of shifting the circadian phase of the SCN. Thus, in a standard light-dark cycle with testing throughout the cycle, it is impossible to completely eliminate these confounds. However, photic effects on the circadian rhythm can be largely eliminated by using very dim red light, a common and long-standing practice in studies of circadian function [70–73]. Additionally, assaying clock-gated locomotor rhythms over the course of the memory assay can provide information on the extent of circadian disruption, if any, caused by the memory assay (as shown in Gritton et al. [12]).

## 2. Circadian Timing Mechanisms

At the cellular level, mammalian circadian rhythms are generated by a self-sustaining transcription/translation feedback loop. At its most fundamental level, this loop is centered on a basic helix-loop-helix transcription factor formed by BMAL1 and CLOCK. This heterodimeric transcription factor binds to E-box motifs (CACGTG) found within the 5′ regulatory regions of *Period1* and *Period2 (Per1/2)* and *Cryptochrome 1* and *Cryptochrome 2 (Cry1/2)* genes, thus leading to their transcription. *Per* and *Cry* transcripts are translated, dimerized, and returned to the nucleus, where they inhibit the function of the BMAL1/CLOCK dimer and hence inhibit their own transcription [74, 75]. Precisely timed degradation of PERIOD proteins relieves the repression of the BMAL1 and CLOCK complex and thus allows for a new round of *Per* and *Cry* transcription to occur. The cycling of this feedback loop, which is set to approximately 24 hours, sets the periodicity of the endogenous cellular oscillators. The phasing, periodicity, and amplitude of this molecular rhythm can be influenced by a wide array of intracellular effectors, including inducible kinases, histone deacetylases, phosphatases, and ubiquitin ligases (for reviews, see [76–80]); hence, this clock feedback loop can be influenced by a wide array of changes in the functional state of the cell (e.g., changes in metabolic activity, stress, and in neurons, excitability).

In mammals, circadian timing is a distributed process, with multiple peripheral organ systems and brain regions exhibiting inherent oscillatory capacity [81–83]. However, the phasing and amplitude of these distributed cell populations are set by a single brain region: the paired suprachiasmatic nucleus of the hypothalamus (SCN). The ~10,000 neurons that form the SCN utilize a variety of local paracrine and synaptic output pathways to convey clock time to peripheral oscillator populations in the brain [81, 83–86]. Further, multisynaptic output pathways allow the SCN to drive rhythmic release of endocrine hormones (e.g., melatonin and glucocorticoids) [81, 83–86], which in turn, impart rhythmic control over energy expenditure, metabolic activity, and both immune and stress responses [87–90]. Further, endocrine hormones also affect the functioning of both the SCN clock and peripheral oscillator populations in the brain [91–95].

Within the forebrain, time-keeping capacity has been reported in various regions, including the cortex, hippocampus, and the amygdala [81, 96, 97]. Consistent with this, forebrain neurons appear to express all of the essential genes required to generate cell-autonomous circadian oscillations [96–98]. Notably, the phasing of circadian rhythms varies between forebrain regions that are important for learning and memory. For example, while the hippocampus and prefrontal cortex peak in *Per1* mRNA expression is at the late night, the amygdala peak of *Per1* mRNA expression is at the late day [97]. The phasing of forebrain circadian rhythms is set by the SCN, and several entrainment mechanisms have been described. Along these lines, SCN-driven rhythms of corticosterone release from the adrenal glands have been shown to contribute to hippocampal rhythm phasing [91–93]. Hence, clamping corticosterone levels in mice eliminates hippocampal rhythmic expression of a *period1*-*luciferase* reporter gene [92], and Woodruff et al. observed that the diurnal modulation of hippocampal-dependent fear conditioning extinction was lost in adrenalectomized rats [99]. Additionally, SCN-driven clock-gated neuronal circuits appear to alter the balance of excitatory versus inhibitory synaptic activity in the hippocampus (in particular via GABAergic innervation from the medial septum [10]). This is supported by recent work demonstrating that the spatial memory deficits in behaviorally arrhythmic Syrian hamsters are abolished following injection of pentylenetetrazol, a GABA antagonist [9, 10].

## 3. Impacts of Circadian Disruption on Memory

Time-of-day gating of hippocampal-dependent memory is dependent in part on the SCN. For example, SCN lesioning (which results in the loss of circadian rhythmicity) causes deficits in long-term novel object recognition [42], contextual fear conditioning and Morris water maze performance [100]. However, no effect of SCN lesioning was observed on performance in intermediate-term novel object recognition [42, 100]. Interestingly, Fernandez et al. [101] found that while an arrhythmic Siberian hamster model had deficits in both working memory (spontaneous alternation) and long-term memory (novel object recognition), ablation of the SCN rescued both forms of memory. In a related line of work, pharmacological GABA inhibition restored performance on the novel object recognition task in arrhythmic Siberian hamsters [9, 10]. This result was used to argue that circadian dysregulation impairs memory by increasing GABAergic inhibition influence within the hippocampus [101]. Together, these data reveal that the SCN timing system has complex context-specific effects on both working and long-term memory processes.

As with SCN lesioning, the effects of the targeted germline deletion of core clock genes (e.g., *Bmal1*, *Cry 1/2*, *Clock*, and *Period1/2*) are complex. *Bmal1* is an essential component of the circadian timing system [102], and thus, *Bmal1* knockout (KO) mice are completely arrhythmic [102, 103]. Notably, *Bmal1* KO mice display deficits in habituation to a novel environment [104] and in contextual fear conditioning and Morris water maze performance [103]; however, enhancement of novel object recognition was also detected in *Bmal1* KO mice [103]. Here, it is worth noting that the memory deficits in this model are somewhat difficult to interpret due to the widespread deleterious effects of *Bmal1* deletion. Along these lines, *Bmal1* KO mice exhibit poor overall health, including premature aging, accelerated rates of mortality, reduced body weight, increased overall sleep, loss of reproductive capabilities, disrupted metabolism, cardiomyopathy, and reduced skeletal muscle function [105–110]. Whether or not these phenotypic effects (including the effects on learning and memory) are related to a loss of circadian timing, or may also result from a loss of BMAL1 transcriptional drive that is independent of the circadian timing system, has not been fully elucidated [111].

In contrast with the complex phenotypic effects of the germline deletion of *Bmal1*, the phenotypic effects resulting from the genetic disruption of other core clock genes are less severe. Along these lines, with respect to overt locomotor (wheel running) activity, multiple *Per1* or *Per2* KO mouse lines exhibit a shortened tau (within the 0.5–1.5 hour time range) relative to WT mice [112–114]. Similarly, the *Clock* null line has a shortened tau (0.4 h) [115], whereas the *Clock^Δ19/Δ19^* mutant mouse line exhibits a 4 hr lengthening of tau and often becomes arrhythmic over an extended period in DD [116]. In tests of hippocampal-dependent memory, the *Per1^Brdm1/Brdm1^* mouse loss-of-function line [114] display normal long-term memory in the Morris water maze and contextual fear conditioning [117], whereas a distinct *Per1* KO mouse line (*Per1^ldc/ldc^* [113]) exhibits deficits on working memory in the radial arm maze [55, 96]. *Clock^Δ19/Δ19^* mutant mice [116] display deficient long-term memory in the Morris water maze yet have passive avoidance memory similar to WT controls [118]. *Cry1/2* double KO mice, which are arrhythmic [119], are unable to acquire the time-place learning task (a form of place preference) [120], whereas arrhythmic *Per1^Brdm1/Brdm1^*/*Per2^Brdm1/Brdm1^* double transgenic mice [114] display a learning curve indistinguishable from WT mice in the time-place learning task [121]. Overall, the complex effects of circadian gene deletion on hippocampal-dependent memory may be due in part to the unique role that each gene product plays in the core clock timing loop, the degree or type of circadian phenotype triggered by the gene deletion, and the extent to which compensatory mechanisms may be recruited to offset the effects of the gene disruption.

Recently, our lab [20] and others [42] investigated the role of non-SCN cell-autonomous circadian oscillations using conditional forebrain *Bmal1* KO models, where *Bmal1* is deleted in a subset of forebrain excitatory neurons (including frontal cortex and hippocampus [20, 42], but excluding the hypothalamus, and hence the SCN [20, 42]). This approach specifically eliminates ancillary clocks without impacting the master SCN clock, thus facilitating experiments addressing the role of ancillary oscillators. In these conditional *Bmal1* KO mice, locomotor rhythms were indistinguishable from WT locomotor rhythms [20, 42], indicating that the functionality of the SCN clock was not affected. The physical health of these animals, notably including gross hippocampal morphology, appears unaffected by conditional *Bmal1* deletion [20]. Importantly, conditional *Bmal1* KO mice exhibit both a total loss of time-of-day dependent novel object location and deficits in Barnes maze performance [20] as well as abrogation of time-of-day dependent novel object recognition [42]. As the circadian deficits in this model are restricted to forebrain excitatory neurons, the cognitive deficits in these mice support a necessary role of hippocampal cell-autonomous oscillations in learning and memory.

## 4. Rhythms in Kinase Signaling

Rhythmic regulation of kinase pathways appears to play a key role in circadian modulation of learning and memory [18, 42–44]. In particular, the ERK/MAPK pathway has been especially well characterized in studies of both learning and memory [122, 123] and circadian timing mechanisms [73, 124]. An initiating event in the stimulation of the ERK/MAPK pathway is the activation (i.e., GTP loading) of the small GTPase Ras. Once in the GTP-bound form, Ras triggers a series of phosphorylation and cellular translocation events that initiates the sequential activation of Raf, MEK, and then ERK. Once activated, ERK functions as the effector kinase of the pathway, targeting numerous proteins within both the cytoplasm and the nucleus. Notably, in the SCN, the ERK/MAPK pathway is highly responsive to photic stimulation and plays a critical role in light-evoked clock resetting [73, 124, 125]. Much of the phase-shifting effects of the ERK/MAPK pathway appear to be mediated via activation of the transcription factor CREB (cAMP response element binding protein) [73, 126]. CREB, in turn, drives the induction of the core clock gene *Per1* [127, 128]. Given these findings, and the noted work showing that the ERK/MAPK pathway plays an important role in learning and memory [122, 123], it appears that the ERK/MAPK pathway is ideally positioned to serve as a regulator of time-of-day dependent synaptic plasticity in the forebrain.

Interestingly, as in the SCN, several studies have reported peak ERK activation during the daytime in the hippocampus [18, 100, 103] (Table 2). This hippocampal oscillation of ERK phosphoactivation is absent in mice with a lesioned SCN [100], indicating that SCN phase-setting signals are necessary for rhythmic hippocampal ERK/MAPK pathway activity. Additionally, *Bmal1* KO animals do not display an oscillation in pERK [103], further indicating that this rhythm is driven by the circadian timing system. Notably, recent research proposes mechanisms by which the circadian clock may regulate the hippocampal ERK/MAPK pathway. Along these lines, the ERK inhibitor SCOP (suprachiasmatic nucleus circadian oscillatory protein) has been shown to function as a regulator of ERK activity rhythms in the hippocampus [42, 129]. SCOP is a polypeptide that inhibits the ERK/MAPK cascade by sequestering nucleotide-free Ras [130]. When a neuron is activated, leading to an increase in cytoplasmic Ca^2+^, activation of the calcium-dependent protease calpain drives rapid degradation of SCOP. This triggers the release of the nucleotide-free Ras, which rapidly binds to GTP. Ras-GTP then activates the ERK/MAPK cascade.

In the CA1 region of the hippocampus, while total SCOP expression is not regulated by time-of-day, the amount of SCOP localized to membrane rafts (where it binds Ras) is highest at night [42] (Table 2). Shimizu et al. [42] reported that learning-induced ERK activation was higher at night in WT animals, correlating with a peak in SCOP localization to membrane rafts. However, this time-of-day regulation of ERK was absent in SCOP conditional KO animals that lacked SCOP in the hippocampus [42], supporting the hypothesis that circadian gating of inducible ERK activation depends on SCOP.

In another study that examined rhythmic regulation of ERK/MAPK signaling, Rawashdeh et al. reported that PERIOD1 (PER1) enhances the nuclear translocation of pP90RSK during the day [44]. P90RSK is an ERK effector that can phosphorylate CREB [131]. Rawashdeh et al. [44] showed a late day peak in hippocampal CREB phosphorylation that was abrogated in the *Per1^ldc/ldc^* KO line, as well as a reduction in CREB-dependent transcription in cultured HT22 hippocampal cells following RNA knockdown of *Per1*. Moreover, coimmunoprecipitation revealed a physical interaction between pP90RSK and PER1, and inducible nuclear localization of pP90RSK was absent in *Per1^ldc/ldc^* mice [44]. Given the noted mechanism by which PER1 shuttles P90RSK to the nucleus, and in turn regulates CREB activity (and hence gene transcription), one would predict that the lack of Per1 protein in *Per1^ldc/ldc^* mice would impact long-term memory and not working memory [39]. However, *Per1^ldc/ldc^* mice display deficits on the radial arm working memory task [55, 96], and Zuegger et al. found no deficits in *Per1^Brdm1/Brdm1^* loss-of-function mice on long-term memory in the Morris water maze and contextual fear conditioning [117].

Additionally, it is noteworthy that while Shimizu et al. argued primarily for increased inducible phosphorylation of ERK during the night [42], Rawashdeh et al. and Saraf et al. argued for increased downstream effects of the ERK/MAPK pathway during the day [44, 55, 132]. Although the phasing of these ERK regulatory processes is distinct between these two studies, the proposed mechanisms may not necessarily be incompatible. Thus, using the SCN as a reference, one finds that (1) basal ERK/MAPK activity is high in subjective day and low at the subjective night and (2) that ERK/MAPK is *only* responsive to light during the night. These findings indicate that the circadian clock restricts specific mechanisms of ERK/MAPK activation to distinct circadian time domains [73]; additional work will be required to determine whether a similar, time-domain-specific, clock-gating mechanism exist in the hippocampus.

Another kinase posited to function as a clock-modulated regulator of hippocampal plasticity is GSK3*β* [43]. Multiple studies have revealed that GSK3*β* signaling plays a key role in hippocampal-dependent forms of learning and memory [133, 134]. Interestingly, Besing et al. [43] demonstrated that in the CA1 cell layer of the hippocampus, GSK3*β* activity was rhythmic, with a peak occurring during the night. Further, Besing et al. [43] found that the inhibition of GSK3*β* diminished LTP only during the night (this finding is further discussed in Section 5). GSK3*β* is a constitutively active kinase that is inhibited by phosphorylation at Serine 9. Although the upstream mechanism that imparts rhythmicity onto GSK3*β* activity is not yet clear, Besing et al. [43] speculate that phosphorylation at Serine 9 by the kinase Akt could underlie the rhythm in GSK3*β* activity in the hippocampus. Notably, expression of the *Akt2* transcript is regulated by the circadian clock in the liver [135], and phosphorylation of Akt follows a circadian rhythm in cardiomyocytes [136].

At the level of clock-gated cellular timing, GSK3*β* has been found to affect the core clock transcriptional loop. In the hippocampus, both BMAL1 rhythms and *period2*-*luciferase* reporter rhythms were disrupted in knock-in mice with constitutively high GSK3*β* activity [43]. Mechanistically, GSK3*β* has been shown to phosphorylate BMAL1, which led to an accelerated rate of degradation [137]. Interestingly, in the liver, Akt has been shown to phosphorylate BMAL1, leading to its increased cytoplasmic localization and, in turn, a decrease in its transcriptional activity [138]. Together, these data indicate that an Akt/GSK3*β* signaling cassette may function at multiple points, both within the core clock feedback loop and within clock-gated processes to modulate neuronal plasticity over the 24-hour cycle.

Finally, Saraf et al. [132] reported that the circadian clock modulates the activation state of mTOR (mammalian target of rapamycin), a key regulator of inducible mRNA translation in the hippocampus. Interestingly, timed daily inhibition of mRNA translation via the injection of anisomycin markedly reduced the efficiency of contextual fear recall [132], thus raising the prospect that circadian gating of mRNA translation plays a critical role in long-term memory persistence or retrieval.

## 5. Long-Term Potentiation and the Circadian Clock

Long-term potentiation (LTP) is a form of synaptic plasticity that is an underlying element in the formation and maintenance of a wide range of memory processes [39, 139, 140]. LTP can be subdivided into two stages: early LTP (E-LTP) and late LTP (L-LTP). Each stage of LTP contributes to distinctive memory processes. E-LTP occurs within the first hour of stimulation and potentiates synaptic strength in early, intermediate-term, and long-term memory by trafficking of glutamate receptors to the postsynaptic membrane [39, 51]. L-LTP, occurring hours poststimulation, is important for intermediate-term and long-term memory and requires gene transcription and translation for maintenance [39].

The population spike (PS) amplitude, duration, and rate of decay of LTP have all been shown to vary based on time-of-day in mice [43, 141–143]. Chaudhury et al. [143] demonstrated that PS amplitude and field excitatory postsynaptic potential (fEPSP) slope were increased in mouse hippocampal slices that were obtained in subjective night, compared to the day. Further, Chaudhury et al. [143] reported that there was a longer duration of the enhanced postsynaptic response in the subjective night compared to day. Interestingly, the enhanced PS amplitude and fEPSP slope during subjective night was consistent between mice kept on a 12-hour light/dark schedule and those kept in constant darkness [143], indicating that this component of LTP is driven by a circadian clock-gated mechanism. Of note, other studies have reported more efficient LTP induction in the subjective day in rats [144] and Syrian hamsters [145]; therefore, there are likely to be species-specific mechanisms by which the clock regulates LTP induction over the 24-hour cycle. Furthermore, germline *Bmal1* knockout mice showed decreased LTP amplitude during the day compared to wild-type animals [103]. Of note, to date, no work has shown if the diurnal difference in LTP PS amplitude or fEPSP slope is abolished in *Bmal1* knockout mice.

Recent work has identified several kinase signaling pathways that may contribute to time-of-day dependent changes in LTP. One such diurnally regulated kinase is GSK3*β*. Notably, increased GSK3*β* activity was found to be correlated with enhanced LTP at night, and the inhibition of GSK3*β* diminished LTP only during the night [43]. Interestingly, in a constitutively active GSK3*β* knock-in mouse, overall LTP was enhanced and was still higher at night than during the day. While the downstream pathway by which GSK3*β* modulates LTP has not been demonstrated, GSK3*β* is known to phosphorylate a range of targets involved in neuronal function and memory, including CREB, tau, *β*-catenin, MAP1B, and PS-1 [146].

Given that circadian modulation of LTP was still observed in the constitutively active GSK3*β* knock-in mouse [43], GSK3*β* is likely not the only pathway regulating time-of-day dependent changes in LTP. Notably, activation of ERK (another kinase diurnally regulated in the hippocampus [18]) has also been shown to play a critical role in induction of early LTP [123] and for the maintenance of L-LTP. The effects on E-LTP may be ascribed to ERK regulation of the Kv4.2 channel, which leads to a reduction in its conductance properties, and in turn, an increase in hippocampal cellular excitability [147, 148]. The effects of MAPK signaling on L-LTP have been shown to result from ERK-dependent activation of CREB-dependent transcription [123, 149]. Given the key role that ERK/MAPK signaling plays in memory formation coupled with noted studies showing ERK activation rhythms in the hippocampus, it is quite tempting to posit that these daily oscillations in ERK activity could contribute to time-of-day dependent changes in learning and memory formation. As a starting point, however, no study to date (to our knowledge) has demonstrated a direct link between the rhythmic regulation of ERK activity and the clock-gating of LTP.

Though work to date indicates that kinase pathways contribute to circadian modulation of intermediate and long-term memory, there is a distinct lack of evidence at the molecular level as to how *working memory* is modulated by the clock (see Table 1 for a summary of circadian differences in working memory). Here, we provide several mechanisms by which this process could occur. One possible mechanism is that the clock gates E-LTP induction, which in turn, could underlie time-of-day differences in working memory efficiency. At a mechanistic level, clock-gated changes in glutamate receptor trafficking efficiency may underlie this process. As noted, E-LTP induction is dependent on the rapid trafficking of AMPA receptors containing the GluA1 subunit to the postsynaptic membrane [51, 150], and recent work has demonstrated that glutamate receptor trafficking is regulated by clock-gated signaling pathways. Notably, Ras-GTP (a kinase in the ERK pathway) has been shown to induce trafficking of GluR1 to synapses [151], and GSK3*β* promotes membrane localization of NMDA receptors [152]. While circadian rhythms in glutamate receptor trafficking have not been reported, this may be a worthwhile area of inquiry. Another possibility is related to clock modulation of dendritic spine density. In support of this idea, Ikeda et al. [153] reported an oscillation in CA1 synaptic spine density, with a peak density occurring during the early subjective night, a time point when peak performance on working memory tasks is observed (see Table 1).

## 6. Conclusions

While the emphasis of this review is on clock gating of excitatory neuronal plasticity, it is important to place these processes within a broader hippocampal context. Notably, the hippocampus is a complex cellular environment with multiple cell types, including excitatory pyramidal cells, inhibitory interneurons, microglia, astrocytes, and oligodendrocytes [154]. Both glia [98, 155] and interneurons [156] have intrinsic circadian rhythms, and both cell types impact learning and memory [157–159]. Further, recent evidence has demonstrated that astrocytes are crucial for setting circadian timing within the SCN [160, 161]. Thus, the work described here, which focuses largely on excitatory neurons, may only be a piece of a much larger intercellular network of neurons and glia through which the clock modulates cognition.

Clearly, a deeper understanding of how the circadian clock gates hippocampal circuitry should be a priority for further study. Potential new avenues of inquiry could include, for instance, synaptic scaling, a process by which neurons regulate their overall level of excitability [66, 162, 163]. While much of the research on synaptic scaling has focused on the effects of sleep, circadian rhythms likely also play a part in this process [164–166]. Further, work that explores the potential impacts that cognitive or neurodegenerative disorders have on clock gating of kinase signaling or clock gating of synaptic plasticity may provide clues regarding the relationship between disease states and deficits in learning and memory. Finally, given the tight, intertwined, relationship between sleep and circadian timing, further experimentation that explores the relative contribution of each process to the modulation of cellular plasticity and cognition is highly merited. Clearly, we are at the beginning of a new and exciting era of work that will provide fundamental insights into the powerful and far-reaching effects that the circadian timing system has on cognition.

## Figures and Tables

**Table 1:** Hippocampus-dependent memory tasks regulated by time-of-day, sorted by memory process (WM: working memory; ITM: intermediate-term memory; LTM: long-term memory; Acq: acquisition; Ret: retrieval).

Process	Assay	Phase (peak/nadir)	Model	Reference
WM	Radial arm maze	Night (21/7)	Sprague–Dawley rats	Hauber and Bareiss [3]
WM	Radial arm maze	Day (2/14)	C3H/HeN mice	Rawashdeh et al. [55]
WM	Spontaneous alternation	Night (19/7)	Siberian hamsters	Ruby et al. [10]
WM	Sustained attention task	Night (16/4)	Sprague–Dawley rats	Gritton et al. [12]
ITM	Novel object location	Night (20/8)	Wistar rats	Takahashi et al. [14]
ITM	Novel object location	Night (16/4)	C57BL/6 mice	Snider et al. [20]
ITM	Novel object recognition	Night (19/3)	Siberian hamsters	Ruby et al. [9]
LTM	Alley maze	Night (18/6)	C57BL/6 Ola mice	Hoffmann and Balschun [2]
LTM	Contextual fear conditioning	Night (14/2)	C57BL/6J mice	Valentinuzzi et al. [19]
LTM	Contextual fear conditioning	Day (4/16)	C57BL/6 mice	Eckel-Mahan et al. [18]
LTM (Ret)	Contextual fear conditioning	Day (3/21)	C57BL/6J and C-3H mice	Chaudhury and Colwell [4]
LTM	Passive avoidance	Day (6/18)	Sprague–Dawley rats	Davies et al. [1]
LTM	Trace fear conditioning	Day (6/18)	C57BL/6 mice	Wang et al. [5]
LTM	Morris Water Maze	Night (16/4)	Sprague–Dawley rats	Gritton et al. [12]
LTM	Radial arm maze	Night (21/7)	Sprague–Dawley rats	Hauber and Bareiss [3]
LTM (Acq)	Novel object recognition	Night (16/4)	C57BL/6 mice	Shimizu et al. [42]

**Table 2:** Memory-related cellular signaling proteins or second messengers found to exhibit rhythmic expression or activity in the hippocampus.

Protein	Region	Method	Phase (peak/nadir)	Model	Reference
cAMP	Whole hippocampus	ELISA	Day (8/20)	C57BL/6 mice	Eckel-Mahan et al. [18]
cAMP	Whole hippocampus	ELISA	Day (4/16)	C57BL/6 mice	Wardlaw et al. [103]
K-Ras	CA1 membrane rafts	Western	Night (16/4)	C57BL/6 mice	Shimizu et al. [42]
4EBP1	Whole hippocampus	Western	Day (4/16)	C57BL/6 mice	Saraf et al. [132]
Akt	Whole hippocampus	Western	Day (4/16)	C57BL/6 mice	Saraf et al. [132]
CREB	Whole hippocampus	Western	Day (4/16)	C57BL/6 mice	Eckel-Mahan et al. [18]
CREB	Whole hippocampus	Western	Day (2/18)	C3H/HeN mice	Rawashdeh et al. [55]
eIF4E	Whole hippocampus	Western	Day (4/16)	C57BL/6 mice	Saraf et al. [132]
ERK	CA1	IHC	Day (4/16)	C57BL/6 mice	Phan et al. [100]
ERK	Whole hippocampus	Western	Day (2/14)	C3H/HeN mice	Rawashdeh et al. [55]
ERK	Whole hippocampus	Western	Day (4/16)	C57BL/6 mice	Wardlaw et al. [103]
ERK	CA1	IHC	Night (16/4)	C57BL/6 mice	Shimizu et al. [42]
ERK	Whole hippocampus	Western	Day (4/16)	C57BL/6 mice	Eckel-Mahan et al. [18]
GSK3*β*	CA1	Western	Day (9/17)	C57BL/6 mice	Besing et al. [43]
mTOR	Whole hippocampus	Western	Day (4/16)	C57BL/6 mice	Saraf et al. [132]
S6	Whole hippocampus	Western	Day (4/16)	C57BL/6 mice	Saraf et al. [132]
Ras-GTP	Whole hippocampus	Western	Day (8/20)	C57BL/6 mice	Eckel-Mahan et al. [18]
SCOP	CA1 membrane rafts	Western	Night (16/4)	C57BL/6 mice	Shimizu et al. [42]
